# Evaluation of MAGE-1 and MAGE-3 as tumour-specific markers to detect blood dissemination of hepatocellular carcinoma cells

**DOI:** 10.1038/sj.bjc.6600016

**Published:** 2002-01-07

**Authors:** D-C Mou, S-L Cai, J-R Peng, Y Wang, H-S Chen, X-W Pang, X-S Leng, W-F Chen

**Affiliations:** Center of Hepatobiliary Surgery, People's Hospital, Peking University Health Science Center, 42 Beilishilu, Beijing 100044, China; Institute of Hepatology, People's Hospital, Peking University Health Science Center, 42 Beilishilu, Beijing 100044, China; Department of Immunology, School of Basic Medical Sciences, Peking University Health Science Center, 38 Xue Yuan Road, Beijing 100083, China

**Keywords:** circulating tumour cells, hepatocellular carcinoma, MAGE transcripts, nested polymerase chain reaction, tumour-specific marker

## Abstract

The members of MAGE gene family are highly expressed in human hepatocellular carcinoma (HCC). In the present study, we tested the tumour-specific MAGE-1 and MAGE-3 transcripts in the peripheral blood of HCC patients by nested RT–PCR to detect the circulating tumour cells and evaluate their potential clinical implication. Of 30 HCC patients, the positive rate of MAGE-1 and MAGE-3 transcripts was 43.3% (13 out of 30) and 33.3% (10 out of 30) in PBMC samples, whilst the positive rate was 70% (21 out of 30) and 53.3% (16 out of 30) in the resected HCC tissue samples, respectively. The positivity for at least one MAGE gene transcript was 63.3% (19 out of 30) in PBMC samples of HCC patients and 83.3% (25 out of 30) in the resected HCC tissue samples. MAGE-1 and/or MAGE-3 mRNA were not detected in the PBMC of those patients from whom the resected HCC tissues were MAGE-1 or MAGE-3 mRNA negative, nor in the 25 PBMC samples from healthy donors. The detection of MAGE transcripts in PBMC was correlated with the advanced stages and tumour size of the HCC, being 82.4% (14 out of 17) in tumour stages III and IVa, 56.6% (five out of nine) in stage II, and null (nought out of four) in stage I. The serum α-FP in 33.3% (10 out of 30) of HCC patients was normal or slightly elevated (<40 ng ml^−1^). However, six of these 10 patients (α-FP <40 ng ml^−1^) were MAGE-1 and /or MAGE-3 mRNA positive in their PBMC. The follow-up survey of MAGE mRNA in PBMC was performed in 12 patients. Seven patients with persistent MAGE-1 and/or MAGE-3 mRNA positive or from negative turned to positive died because of metastasis and/or recurrence. In striking contrast, all four patients with MAGE-1 and/or MAGE-3 mRNA from positive turned to negative and one patient with persistent MAGE-3 transcript negative are alive after last test. Collectively, detection of MAGE transcripts with follow-up survey in PBMC is a feasible and reliable assay for the early prediction of the relapse and prognosis of the HCC patients.

*British Journal of Cancer* (2002) **86**, 110–116. DOI: 10.1038/sj/bjc/6600016 www.bjcancer.com

© 2002 The Cancer Research Campaign

## 

Hepatocellular carcinoma (HCC) ranks among the most common malignancies in China, Japan, Southeast Asia, South Africa and some South European areas. Though regular sonographic examination and serum alpha-fetoprotein (α-FP) can detect small HCC at an early stage and there are many modalities of treatment, the recurrence and metastasis are frequent and the prognosis remains unsatisfactory. The high recurrence rate is probably attributed to the dissemination of HCC cells into blood circulation. Early detection of metastatic tumour cells is critical to identify HCC patients at high risk of relapse and for the prescriptive therapy. However, it is difficult to detect such dissemination of HCC cells through blood route with conventional techniques.

The reverse transcription-polymerase chain reaction (RT–PCR) has made it possible to detect molecular markers present at low copy numbers for the evaluation of micro-metastasis. In 1991, Smith and colleagues (Smith *et al*, 1991) first successfully adopted RT–PCR technique to assess tyrosinase messenger RNA (mRNA) as a tumour marker to detect circulating melanoma cells. Then, the gene transcripts of both tissue-specific and tumour specific markers have been applied in RT–PCR based diagnosis of micrometastasis of tumour cells in peripheral blood, such as the tyrosinase and MAGE-3 transcripts in melanoma (Brossart *et al*, 1993; Foss *et al*, 1995; Hoon *et al*, 1995; Smith *et al*, 1991), prostate specific antigen (PSA) and prostate specific membrane antigen (PSMA) encoding gene transcripts in prostate cancer (Israeli *et al*, 1994, 1995), carcinoembryonic antigen (CEA) in colon cancer (Mori *et al*, 1996), CD44 and CK-19 transcripts in breast cancer (Dall *et al*, 1995; Kruger *et al*, 1996) and tyrosinase hydroxylase in neuroectodermal tumour (Miyajima *et al*, 1996; Naito *et al*, 1991). With respect to HCC, both albumin and α-FP mRNA are widely used as tumour markers for HCC cells in circulation (Chou *et al*, 1994; Hillaire *et al*, 1994; Jiang *et al*, 1997; Kar and Carr, 1995; Komeda *et al*, 1995; Lemoine *et al*, 1997; Malek *et al*, 1999; Matsumura *et al*, 1994; Wong *et al*, 1997). However, the reliability using them as tumour markers have been challenged, because both albumin and α-FP are abundantly expressed in normal liver cells, they are released to the peripheral blood by either surgical injury of the liver for the disease other than HCC or by hepatitis virus infection (Chou *et al*, 1994; Jiang *et al*, 1997; Lemoine *et al*, 1997; Malek *et al*, 1999; Wong *et al*, 1997). It is, therefore, necessary to screen tumour-specific markers, which are specifically and frequently expressed in HCC.

Since 1991, MAGE-1 (melanoma associated antigen) gene has been cloned from melanoma cells (van der Bruggen *et al*, 1991), there are up-to-date 17 MAGE genes being cloned (De Plaen *et al*, 1994; Lucas *et al*, 1998; Lurquin *et al*, 1997; Muscatelli *et al*, 1995; van der Bruggen *et al*, 1991). The MAGE genes are activated in spermatozoa but silent in normal somatic cells. In cancers, these genes are re-activated and their encoding proteins are frequently expressed in various histological types of cancers. Because of this distribution characteristic, the MAGE proteins are termed as cancer-testes (CT) antigens (De Plaen *et al*, 1994). Both others and our group have reported that MAGE-1 and MAGE-3 transcripts were highly expressed in HCC tissues, but not in non-HCC liver tissues, nor in non-HCC liver diseases such as HBV/HCV infection and cirrhosis (Cai *et al*, 1999, 2000; Chen *et al*, 1999; Tahara *et al*, 1999; Yamashita *et al*, 1996). Therefore, we investigated if the MAGE gene transcripts could be used as tumour marker to specifically detect metastasis of HCC cells in peripheral blood. We have developed a nested RT–PCR assay to detect MAGE-1 and MAGE-3 transcripts in PBMC, through which to evaluate them as the early markers to unveil hematogeneous dissemination of HCC cells and their potential clinical implications.

## MATERIALS AND METHODS

### Cell lines and human tissues

Mel-Ed1, ME235, 2.2ETI (three melanoma cell lines were kindly provided by Dr Qi-Yuan Chen from Ludwig Institute for Cancer Research, Melbourne Tumour Biology Branch, Melbourne, Australia) grown in Dulbecco's modified eagle medium with 10% foetal calf serum were served as positive controls for the expression of MAGE-1 and MAGE-3 gene transcripts. Samples of HCC tissues and adjacent non-cancerous tissues were collected from HCC patients (*n*=30) who underwent hepatectomy at either the 2nd or 8th Hospitals of Peking University Health Science Center. All samples were frozen in liquid nitrogen until RNA extraction. Informed consent was obtained from each patient before the study was conducted. The Ethic Committee of the University approved the study protocol. The stage of HCC was determined according to the criteria outlined by the Liver Cancer Study Group of Japan (1989). The clinical diagnosis was confirmed by pathological examination.

### Blood samples

Whole blood samples were drawn on the day before surgery from these 30 HCC patients. Control blood samples were collected from 25 healthy donors. Approximately 20 ml of blood from each patient were collected in two tubes, 10 ml in heparinized tube for the separation of peripheral blood mononuclear cells (PBMC), and another 10 ml in plain tubes for the separation of serum to detect serum α-FP. For PBMC separation, 10 ml blood was diluted with 10 ml RPMI1640 medium containing 10% foetal bovine serum and placed on a Ficoil-Hypaque layer (GIBCO BRL, USA), and the PBMC were collected after density gradient centrifugation.

### RT–PCR

Total RNA was extracted from frozen tissue specimens (50–100 mg) and freshly isolated PBMC using TRIZOL (GIBCO BRL) reagent according to the protocol provided by the manufacturer. Total RNA (2.5 μg) was primed with an Oligo(dT)15 oligonucleotide (Promega) and reverse-transcribed with Superscript II (GIBCO BRL) according to manufacturer's instructions. The amplification reaction (50 μl) contained 1 μl cDNA, 2.5 μl each of 10 μM primers and 2.5 U Taq polymerase (GIBCO BRL) in buffer solution. The PCR amplifications were performed by pre-programmed Thermal Cycler (Perkin-Elmer, USA) under the following conditions: (1) cDNA from liver tissues: The samples were denatured at 94°C for 4 min followed by the amplification of MAGE-1 (94°C for 45 s, 65°C for 45 s, and 72°C for 45 s) and MAGE-3 (94°C for 45 s and 72°C for 3 min) for 35 cycles. The sequence of MAGE-1 and MAGE-3 primers and length of PCR products were as follows: MAGE-1: forward-5′-CGG CCG AAG GAA CCT GAC CCA G-3′ (CHO-14) and reverse-5′-GCT GGA ACC CTC ACT GGG TTG CC-3′ (CHO-12), size, 421 base pair (bp); MAGE-3: forward-5′-TGG AGG ACC AGA GGC CCC C-3′ (AB-1197) and reverse-5′-GGA CGA TTA TCA GGA GGC CTG C-3′ (BLE-5), size, 725 bp. (2) cDNA from PBMCs: The reaction conditions for the first round of PCR were completely the same as those for PCR amplification of cDNA in liver tissues, but were carried out just for 25 cycles. For the second round of PCR, 5 μl of a 1 : 10 dilution of the first-round PCR product was used in combination with 2.5 μl each of 10 μM MAGE-1 (M1nestX: forward-5′-ACA GAG GAG CAC CAA GGA GAA G-3′; M1nestY: reverse-5′-AGT TGA TGG TAG TGG GAA AGG C-3′, size, 299 bp) or MAGE-3 (M3nestX: forward-5′-CGG AGG AGC ACT GAA GGA GAA G-3′; M3nestY: reverse-5′-CCT CCT CTT CTT GGT TGC TGG-3′ size, 371 bp) inner primers, which were designed with Gene Runner analysis software 3.04 (Hastings Software Inc.). After initial heating at 94°C for 2 min, 35 cycles of PCR were then carried out for the amplification of MAGE-1 (94°C for 45 s, 65°C for 45 s, and 72°C for 45 s) and MAGE-3 (94°C for 45 s, 69°C for 45 s, and 72°C for 45 s). (3) To assess the integrity of the cDNA, β-actin (CHO15: forward-5′-GGC ATC GTG ATG GAC TCC G-3′ and CHO16: reverse-5′-GCT GGA AGG TGG ACA GCG A-3′, size, 613 bp) was amplified for 28 cycles (45 s at 94°C, 55°C and 72°C separately). For analysis, 6 μl of reaction product was run in 2% agarose gel (Promega, USA), followed by ethidium bromide staining and digital camera photographing.

### Cloning and sequencing of MAGE cDNA

Purified MAGE-1 and MAGE-3 cDNA from PCR amplification was cloned into pGEM-T Easy Vector (Promega) by T4 DNA ligase and amplified in *E. coli*, JM105. The positive colonies were selected using *Eco*RI digestion of mini-prepared DNA. The putative MAGE cDNA samples were sequenced with T7 sequencing primers in Saibaisheng Co., Beijing, China.

### Follow-up

Follow-up was obtained from all the patients every 3–6 months after operation. All the patients were checked with ultrasonography, computed tomography (CT) and serum α-FP to confirm if there were any metastasis and/or recurrence. If possible, whole blood samples were drawn to get PBMC, then MAGE-1 and MAGE-3 were amplified to find out any changes in expression of the two MAGE genes in PBMC, according to the methods as described above. The endpoint of follow-up was set at the death of the patient.

### Statistical analysis

The statistical analysis was performed by the Fisher's exact probability test because the samples were small in number. The level of significance was set at *P*<0.05.

## RESULTS

### Patients

A total of 30 HCC patients were enrolled in the study. All the patients had chronic HBV infection with various degrees of cirrhosis. Two of the patients were complicated with HCV infection. In all the normal controls, there was no sign of hepatitis or liver functional abnormality. Pathological examination indicated that 16.7% (5 out of 30) of HCC samples were well differentiated, 46.7% (14 out of 30) moderately and 36.7% (11 out of 30) were poorly differentiated, respectively. Twenty-four (80%) patients had large sized tumours (⩾4 cm), while the tumour diameter of the other six patients (20%) was less than 4 cm. According to the TNM classification of the tumour, there were 13.3% (4 out of 30) at stage I, 30% (9 out of 30) at stage II, 30% (9 out of 30) at stage III, and 26.7% (8 out of 30) at stage IVa, respectively (Table 1

**Table 1:**
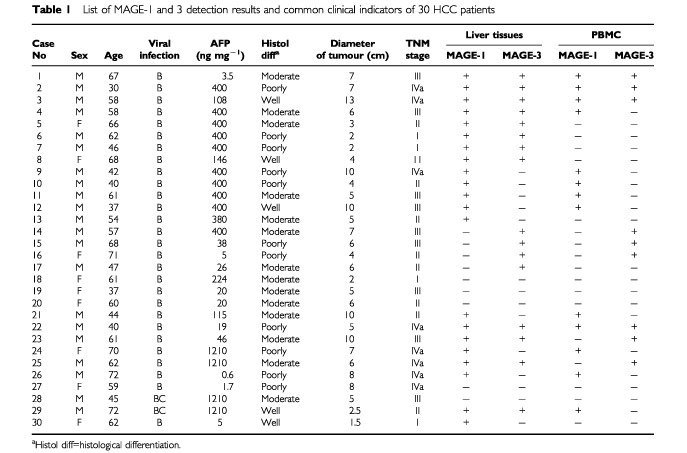
List of MAGE-1 and 3 detection results and common clinical indicators of 30 HCC patients

).

### Sensitivity of the nested RT*–*PCR technique

**Figure 1:**
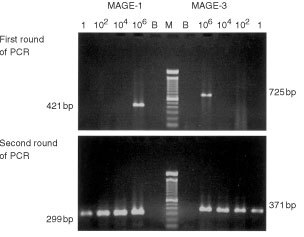
cDNA derived from 1×10^7^ Mel-Ed1 cells was 100-fold sequentially diluted. After 25 cycles of the first round RT–PCR amplification, MAGE-1 and MAGE-3 transcripts were detectable only in the cDNA of 10^6^ cells and shown in bands of 421 and 725 bp individually. After the second round of 35 cycles of amplification, MAGE-1 (299 bp) and MAGE-3 (371 bp) transcripts were detected in the PCR products amplified from the cDNA converted from one cell contained mRNA.

To determine the sensitivity of the method, 100-fold serial dilution experiments were performed using cDNA from 10^7^ Mel-Ed1 cells that express both MAGE-1 and MAGE-3 transcripts. The cDNA dilutions of 1/10 (10^6^ cells), 1/10^3^ (10^4^ cells), 1/10^5^ (10^2^ cells), 1/10^7^ (one cell) were utilized as templates for two rounds of RT–PCR amplification of MAGE-1 and MAGE-3 transcripts. After the first round (25 cycles) of PCR amplification, only at the dose of cDNA from 10^6^ cells were both MAGE gene transcripts detected. However, after the second round (35 cycles) of PCR amplification, the two MAGE genes could be sensitively and clearly detected from the cDNA contained in about one Mel-Ed1 cell (Figure 1

).

### Nested RT*–*PCR results

**Figure 2:**
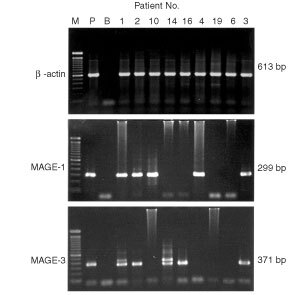
Electrophoresis of second round of PCR products amplified from cDNA of PBMC samples of HCC patients. (1) M: molecular marker, 100 bp DNA ladder (Gibco); (2) P: Mel-Ed1, Positive control of nested MAGE-1 (299 bp) and MAGE-3 (371 bp) transcripts; (3) B: Blank control, PCR amplification in the absence of template; (4) β-actin (613 bp), cDNA quality control, which was amplified for one round of 35 cycles and all the samples shown here were positive; (5) Patient No. 1, 2, 3, 4, 10 showed MAGE-1 transcript positive, while patient No. 1, 2, 3, 14, 16 showed MAGE-3 transcript positive: (6) The second band of MAGE-3 transcript shown in Patient No. 1 and 14 was the genomic DNA with the size of 452 bp.

With two-round RT–PCR amplification, the positive rate in blood samples was 43.3% (13 out of 30) for MAGE-1 transcript and 33.3% (10 out of 30) for MAGE-3 transcript. 63.3% (19 out of 30) of the PBMC samples were positive for at least one type of the two MAGE gene transcripts (Table 1). Among 25 cases of MAGE-1 and/or MAGE-3 mRNA positive HCC tissue samples, at least one of the two MAGE transcripts was detected in the PBMC of 19 cases of patients. The correlation positive rate was 76% (19 out of 25) between two distinct sources of specimen. MAGE-1 or MAGE-3 transcript was not detected in the PBMC of the patients whose resected HCC tissue samples were negative for MAGE-1 or MAGE-3 mRNA. In control samples, MAGE-1 or MAGE-3 mRNA could not be detected in PBMC from 25 healthy donors. The MAGE-1 and MAGE-3 positive PCR products amplified from mRNA of the PBMC samples were cloned into competent bacteria and three clones from each were randomly picked up and sequenced. The nucleotide sequence of MAGE-1 or MAGE-3 cDNA fragments were identical to the database of the GenBank. It confirms that the RT–PCR products were truly MAGE-1 and MAGE-3 cDNA (data not shown). The typical electrophoresis of nested RT–PCR products amplified from cDNA of PBMC samples of some HCC patients is shown in Figure 2

.

### RT*–*PCR status and clinical indicators

The frequency of MAGE-1 or MAGE-3 transcript positive cases detected in PBMC of patients with HCC at different stages was: null (0 out of 4) at stage I, though 3 out of 4 of resected HCC tissue samples were positive for at least one of the two MAGE transcripts, 56.6% (5 out of 9) at stage II, 77.8% (7 out of 9) at stage III, and 87.5% (7 out of 8) at IVa. Taken the expression of MAGE transcripts in HCC tissues into account, the correlation rate of MAGE-1 or -3 transcript expressed in HCC tissue samples and detected in PBMC samples was 62.5% (5 out of 8) at stage II, 100% (7 out of 7) at stage III, and 100% (7 out of 7) at IVa. Of note, MAGE-1 or MAGE-3 mRNA was detected in PBMC in 38.5% (5 out of 13) patients with the HCC at early stages (stages I and II). MAGE-1 or -3 transcript could be detected in virtually all PBMC samples of the 14 patients with HCC at stage III or IVa from whom the resected HCC tissue samples were all positive for these genes. Three of the 17 patients bearing HCC of stage III or IVa were MAGE-1 or -3 mRNA negative in both resected HCC tissues and PBMC. Regarding the size of the cancer, 75% (18 out of 24) and 16.6% (1 out of 6) of blood samples were MAGE-1 or -3 mRNA positive in the patients with large sized tumours (⩾4 cm) and small sized tumours (<4 cm), respectively. Clearly, the frequency of the MAGE transcripts detected in PBMC is in strong correlation with clinical severity. The frequency of the MAGE transcripts detected in PBMC was significantly higher in HCC patients at stages III and IVa than at stages I and II (*P*=0.018) and in large tumours than in small tumours (*P*=0.009). The expression of MAGE-1 or -3 transcripts did not correlate with any of the other parameters, such as the degree of tumour differentiation, serum α-FP, etc (as listed in Table 1).

### α-FP level did not correlate with MAGE transcripts in blood samples of HCC patients

The cases of α-FP level (40 ng ml^–1^) in the serum of patients with HCC were 3 out of 4 in stage I, 6 out of 9 in stage II, 6 out of 9 in stage III, and 5 out of 8 in stage IVa. There is no correlation between the α-FP level and the stages of the cancer. The overall positive rate of α-FP in serum was 66.7% (20 out of 30) and of MAGE-1 and/or MAGE-3 mRNA in PBMC samples was 63.3%. There was no correlation between the positivity of α-FP and MAGE transcripts in blood samples in each individual HCC patient. However, 33.3% (10 out of 30) of HCC patients had serum α-FP lower than 40 ng ml^−1^. In these 10 patients, six patients had MAGE-1 or MAGE-3 transcript detected in PBMC samples. It is noted that among the resected HCC tissue samples from these 10 patients, the MAGE-1 or -3 mRNA was positive in 6 samples, while negative in another four samples, hence the positive rate of MAGE-1 or -3 mRNA in PBMC samples among α-FP negative patients is underestimated. By contrast, in five patients both resected HCC tissue and PBMC samples were MAGE-1 or -3 mRNA negative, three patients had the α-FP higher than 40 ng ml^−1^. Although there is no correlation between the positive rates of α-FP and MAGE-1 or -3 transcript in blood samples with respect to each individual patient, combination of the parameters of α-FP in serum and MAGE-1 or -3 mRNA in PBMC, a higher positive rate has been achieved (28 out of 30, 93.3%).

### Detectable MAGE transcripts in PBMC and prognosis

All the patients with tumour resection were followed-up for the average time of 13.7 months. Among 19 patients with MAGE-1 and/or MAGE-3 transcripts detected in PBMC, 15 patients died, one died from liver functional failure, the other 14 patients (77.8%) died from recurrence and/or metastasis. The death rate in the patients with undetectable MAGE-1 or MAGE-3 transcript in PBMC was 2 out of 6 (33.3%). Because of the small number of patients under investigation, there was no statistical difference (*P*>0.05) in the death rate or relapse between the two groups of patients distinguished by preoperative single detection of MAGE-1 and MAGE-3 mRNA in PBMC. The perturbation of MAGE-1 and/or -3 transcripts in blood after resection of MAGE-1 and/or -3 positive tumours has been followed up in 12 patients (Table 2

**Table 2:**
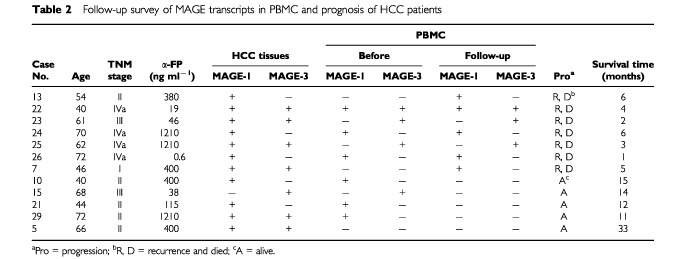
Follow-up survey of MAGE transcripts in PBMC and prognosis of HCC patients

). The MAGE-1 or -3 transcript in PBMC was persistently detectable in five patients and all these five patients died for recurrence and/or metastasis. In two patients (one at stage II, the another at stage I), the MAGE transcripts in PBMC were turned from negative to positive in the following up period, these two patients died of recurrence and/or metastasis. In contrast, among these 12 patients, four patients (three in stage II, one in stage III) showed both clinical stable and MAGE-1 or -3 transcript undetectable in PBMC (i.e., MAGE transcripts from detectable before surgery turned to negative after surgery). All these four patients are alive 14-months, 12-months, 11-months and 15-months after tumour resection, respectively, with no sign of recurrence or metastasis. One patient with persistent negative of MAGE transcripts in PBMC is alive with clinical remission 33 months after tumour resection. Thus, the recurrence and death rate were significantly higher in the patients with persistent detectable, or from undetectable to detectable, MAGE-1 and MAGE-3 mRNA in PBMC than those patients with MAGE transcripts from detectable turned to undetectable, or persistently undetectable, in PBMC (*P*<0.01).

## DISCUSSION

Metastasis spreading through vessels is the most important factor affecting the prognosis of HCC patients. If the HCC cell metastasis via hematogenous route can be sensitively and specifically determined at early stage, more beneficial therapeutic methods could be manipulated. With the advance of molecular biology, the technique of RT–PCR amplification of specific DNA sequences using synthesized oligonucleotide primers that flank the target DNA fragment of interest is increasingly applied in the detection of micrometastasis of cancer cells (Brossart *et al*, 1993; Dall *et al*, 1995; Foss *et al*, 1995; Hoon *et al*, 1995; Israeli *et al*, 1994, 1995; Kruger *et al*, 1996; Miyajima *et al*, 1996; Mori *et al*, 1996; Naito *et al*, 1991; Smith *et al*, 1991). Our results verified exponential amplification of target cDNA converted from mRNA allowed to detect a single malignant cell within millions of normal blood cells and hence, to sensitively detect the metastatic tumour cells in peripheral blood (Negrin and Blume, 1991). Apparently, RT–PCR-based detection of circulating tumour cells is much more sensitive than that diagnosed by antibody-based serology. In this study, we have developed a sensitive and specific technique capable of detecting one single circulating HCC cell in blood samples using MAGE-1 and MAGE-3 transcripts as tumour-specific markers.

Though elevation of serum α-FP has been routinely applied as a parameter for HCC diagnosis, α-FP transcripts were detected in normal liver cells, the liver cirrhosis and the liver infectious diseases such as HBV/HCV infection (Jiang *et al*, 1997). Furthermore, α-FP transcripts was detected even in the blood samples from patients without HCC after the surgical injury of the liver, which results in the shedding of liver cells into circulation under surgical operation (Lemoine *et al*, 1997; Malek *et al*, 1999). Thus, α-FP represents a liver cell-specific marker, not a tumour-specific marker. In HCC, the majority cases of HCC cells produce α-FP leading to its elevation in blood samples and providing an adjunct diagnosis. However, a significant proportion of patients does not have elevated α-FP. We found in this study that 33.3% (10 out of 30) HCC patients with the serum α-FP lower than 40 ng ml^−1^. Our study also showed that the elevation of α-FP is not correlated with the progression of the HCC. Thus, α-FP is valuable to help diagnosis of HCC, but not a specific marker to detect circulating HCC cells.

MAGE gene transcripts have been regarded as tumour-specific markers and found to be highly expressed in a variety histological types of cancers. In the case of HCC, the positive rate was 46–80% of MAGE-1 transcript and 42–68% of MAGE-3 transcript in HCC tissue samples (Cai *et al*, 1999, 2000; Chen *et al*, 1999; Tahara *et al*, 1999; Yamashita *et al*, 1996). A proportion of 74–86% of the HCC tissue samples was positive for at least one MAGE gene transcript, while no expression of MAGE transcripts detected in the surrounding non-cancerous tissues, nor in the livers of cirrhosis, HBV/HCV infection or normal ones (Cai *et al*, 1999, 2000; Chen *et al*, 1999; Tahara *et al*, 1999; Yamashita *et al*, 1996). We have reported previously that MAGE-1 and MAGE-3 transcripts were expressed in 71 and 78% of resected HCC tissue samples, respectively (Cai *et al*, 1999; 2000). In our present study, 83.3% (25 out of 30) of the HCC tissues were positive for either MAGE-1 or MAGE-3 transcript. In conjunction with the prevalent invasion of HCC cells to hepatic vessels, it is logical to consider that MAGE-1 and MAGE-3 could be the appropriate tumour-specific markers for the detection of circulating HCC cells.

With nested RT–PCR assay, 43.3% (13 out of 30), 33.3% (10 out of 30) and 63.3% (19 out of 30) were found to be positive for MAGE-1, MAGE-3, and MAGE-1 and/or MAGE-3 transcripts, respectively, in the peripheral blood samples. Detection of MAGE-1 and/or MAGE-3 transcripts in PBMC is closely correlated to the pathological stages of HCC. The more advanced stages of HCC, the higher rate of micro-metastasis of cancer cells detectable in peripheral blood (to compare the positive rate between the HCC samples in stages III and IVa with those in stages I and II, *P*<0.02). Concerning the three HCC tissue samples in stage III or IVa did not express MAGE-1 or MAGE-3 transcript, virtually all the patients with HCC in stages III and IVa have had cancer cells metastasized to the peripheral blood. According to the TNM criteria, the HCC in stage II (T2NoMo) should have no other metastasis of tumour cells except the possibly intrahepatic metastasis. Assay by nested RT–PCR to detect MAGE-1 and/or MAGE-3 transcripts, the tumour-specific markers, revealed that 56.6% (5 out of 9) patients with HCC in stage II have already had micro-metastasis to the peripheral blood, a parameter to detect occult hematogenous dissemination of HCC cells much earlier than any other means described. This parameter firmly indicates that blood dissemination of tumour cells has already occurred in the early stage of HCC. It is probably the reason why some patients still suffered recurrence after radical resection of the tumour. In terms of actual presence of circulating HCC cells in blood, the TNM defined early stage is inadequate which ignores the blood metastasis and needs to be modified according to the new technology approaches.

The important implication for the detection of occult hematogenous metastasis of HCC cells by nested RT–PCR of MAGE-1 and/or MAGE-3 transcripts is its value in the prediction of recurrence after treatment and prognosis of the disease. In the follow-up survey in 12 patients after tumour resection, five patients with persistent expression of MAGE-1 and/or MAGE-3 transcripts in PBMC samples and two patients with MAGE-1 and/or MAGE-3 transcripts from negative tumour resection turned to positive after resection died due to relapse. In contrast, four patients with MAGE-1 and/or MAGE-3 transcripts in PBMC samples from positive before tumour resection turned to negative after surgical treatment and one patient with persistent negative before and after operation are all alive. The difference between two groups of patients with respect to the recurrence and death rate has statistic significance (*P*<0.01). Apparently, the continuous positive or negative of circulating tumour cells assessed by MAGE transcripts in blood cells is closely correlated to the prognosis. Such follow-up survey is not only a predictor for prognosis of the patients, but also a valuable parameter to judge the effectiveness of the treatment regime employed. For the limited number of patients in our follow-up survey, our correlation date between the status of detectable MAGE transcripts in blood and the prognosis of the patients is preliminary. Quantitative estimation of the number of circulating HCC cells and the probability of the metastasis in secondary sites or reseeding into the liver to cause recurrence needs to be more detailed analyses in more patients and in long-term follow-up surveys.

Just in the period of performing follow-up survey required by referees of the journal, a paper has been published by Miyamoto *et al* (2000). They have used conventional RT–PCR with one-round amplification of MAGE-1 and MAGE-3 transcripts in the blood samples of HCC patients. In no patients could the MAGE transcripts be detected in peripheral blood samples prior to surgical operation. Only after operation could the MAGE transcripts be detected in peripheral blood with very low positivity (1 out of 11 for each MAGE transcripts). They did not do follow-up survey with the MAGE transcript detection and prognosis. However, they detected more MAGE transcripts in the hepatic venous blood and in the portal venous blood prior, during and after operation with overall positive rate of 18.3% (13 out of 71 blood specimens collected from 11 patients), supporting the HCC invasion of the blood vessels localized in the liver organ. Notably, in their 13 blood specimens with positive MAGE transcript, only two specimens were positive for α-FP transcript, consistent with our results.

In summary, detection of α-FP and MAGE transcripts in blood samples has different significance. α-FP is an adjunct assay which may help early diagnosis of HCC, as α-FP is elevated in some patients with HCC in stage I, whereas detection of MAGE transcripts in blood, especially with follow up survey, may help to predict the prognosis and monitoring of the response to the therapy. Combination of both may be more valuable in the diagnosis.
